# Incidence and Risk Factors for Retinopathy of Prematurity at a Rural Tertiary Hospital in Thailand

**DOI:** 10.18502/jovr.v18i1.12728

**Published:** 2023-02-21

**Authors:** Mantapond Ittarat, Supakorn Chansaengpetch, Sunee Chansangpetch

**Affiliations:** ^1^Surin Hospital and School of Ophthalmology, Suranaree University of Technology, Surin, Thailand; ^2^Queen Savang Vadhana Memorial Hospital, Chonburi, Thailand; ^3^Center of Excellence in Glaucoma, Faculty of Medicine, Chulalongkorn University and King Chulalongkorn Memorial Hospital, Thai Red Cross Society, Bangkok, Thailand; ^5^https://orcid.org/0000-0001-8177-1234; ^6^https://orcid.org/0000-0002-8996-2868

**Keywords:** Incidence, Retinopathy of Prematurity, Risk-factors, Thailand

## Abstract

**Purpose:**

To estimate the incidence and identify the factors affecting retinopathy of prematurity (ROP) in a rural tertiary hospital in Thailand.

**Methods:**

This retrospective chart review included all infants screened for ROP. The study included all infants with gestational age (GA) ≤ 30 weeks or birth weight (BW) ≤ 1,500 gr or selected larger infants with an unstable clinical course. Retinal findings were classified according to the revised International Classification of ROP. Data were analyzed using univariate and multivariable logistic regression analyses.

**Results:**

Of the 113 screened infants, the incidences of any ROP and ROP requiring intervention were 17.7% and 8.8%, respectively. In univariate analysis, lower GA, lighter BW, total days of supplemental oxygen, days of continuous positive airway pressure (CPAP), presence of apnea, and intraventricular hemorrhage (IVH) were associated with the development of any ROP. In the stepwise multivariable logistic regression analysis, lighter BW, male gender, and bronchopulmonary dysplasia (BPD) were significant risk factors for the development of any ROP. Lower GA and being either a twin or triplet were significant risk factors for ROP requiring intervention. However, no antenatal condition was identified as a risk factor for ROP.

**Conclusion:**

The incidence of ROP in rural tertiary hospitals was relatively high as compared with previously published data from urban tertiary hospitals. Lighter BW, male gender, and BPD were significantly associated with the development of ROP in a local context. Epidemiological studies are necessary to prevent ophthalmic morbidities.

##  INTRODUCTION

Retinopathy of prematurity (ROP) is one of the leading causes of childhood preventable blindness worldwide. Globally, it was estimated that 184,700 infants developed ROP in 2010.^[1]^ Subsequently 20,000 became blind or suffered from severe visual impairment, which could have been avoided with timely screening and interventions for ROP.^[1,2]^ Many nations are now addressing ROP as one of the major public health concerns.^[3]^

Various factors have demonstrated to be associated with the development of ROP, some of which also influence its severity. Gestational age (GA), birth weight (BW), and use of supplemental oxygen showed the strongest association with ROP.^[4,5]^ Other neonatal comorbidities such as respiratory distress syndrome (RDS), bronchopulmonary dysplasia (BPD), intraventricular hemorrhage (IVH), and necrotizing enterocolitis are also well recognized as relevant risk factors for ROP development.^[5,6,7]^

Recent advances in neonatal care have improved the survival rate of premature infants, leading to an increase in the incidence of ROP.^[3,8,9]^ However, the current situation in developing and Asian countries, including Thailand, is now considered an epidemic.^[2,3]^ The incidence of ROP in any stage varies among different countries. Even in the same country, the incidence and risk factors of ROP differ from region to region, as there are differences in regional ethnicities and characteristics of antenatal and neonatal care between urban and rural areas. According to an urban tertiary hospital in Bangkok, 40.7% of premature infants who underwent screening examinations during 2006–2009 developed ROP.^[10]^ Worse, epidemiological data on ROP in Thailand are still lacking, particularly in rural areas; very few studies on ROP have been published in the past decade. Pinpointing our study to a regional context can provide key information for the further identification and investigation of more specific factors for ROP. Therefore, this study aimed to estimate the incidence of ROP and identify potential risk factors for ROP in a rural tertiary hospital in Thailand.

**Figure 1:**
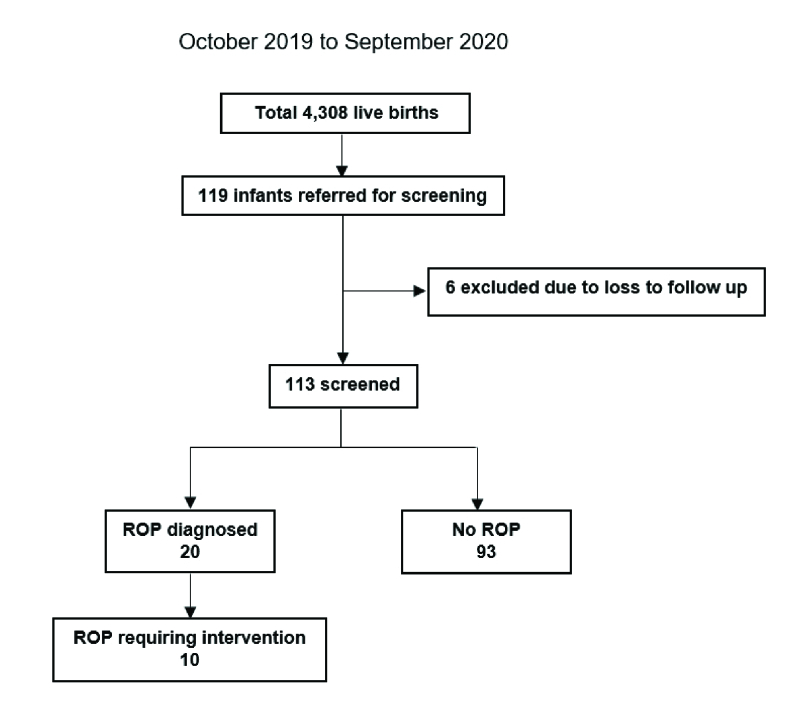
Schematic diagram of study recruitment. ROP, retinopathy of prematurity.

**Table 1:** Univariable analysis – Risk factors for ROP.


	orange**No ROP **	orange**Any ROP **	orange ***P-*** **value ${}^{a}$ **	orange**ROP requiring intervention**	orange ***P-*** **value ${}^{b}$ **
	*N* = 93	*N* = 20	*N* = 10	
Gender: male	45 (48.4)	14 (70.0)	0.079 ${}^{*}$	8 (80.0)	0.065*
Birth weight (gr)	1656.1 (500.9)	1294 (246.8)	0.002 ${}^{†}$	1287 (227.2)	0.037*
Twin/triplet		0.067 ${}^{*}$	0.001 ${}^{§}$
Twin	15 (16.1)	4 (20.0)	4 (40.0) 2 (20.0)	
Triplet	1 (1.1)	2 (10.0)		
**Antenatal factors**			
Gestational age (wk)	32.2 (2.9)	30 (3.2)	0.002 ${}^{†}$	28.7 (1.3)	0.004
Maternal age (yr)	25.4 (7.1)	27.5 (7.9)	0.103 ${}^{†}$	28.7 (8.7)	0.266 ${}^{†}$
Number of gestations	2 (1 to 3)	1 (1 to 2)	0.912 ${}^{‡}$	1 (1 to 4)	0.780 ${}^{‡}$
Inadequate number of ANC visits	2 (2.3)	1 (5.9)	0.446 ${}^{§}$	1 (14.3)	0.192 ${}^{§}$
Maternal diabetes mellitus	4 (4.7)	1 (5.9)	1.000 ${}^{§}$	0 (0.0)	1.000 ${}^{§}$
Premature rupture of membranes	8 (9.3)	1 (5.9)	1.000 ${}^{§}$	0 (0.0)	1.000 ${}^{§}$
Severe preeclampsia	4 (4.7)	0 (0.0)	1.000 ${}^{§}$	0 (0.0)	1.000 ${}^{§}$
**Neonatal factors**			
1-min Apgar score	7.2 (2.4)	6.6 (3.0)	0.382 ${}^{†}$	7.1 (1.7)	0.953 ${}^{†}$
5-min Apgar score	8.8 (2.0)	8.4 (2.8)	0.502 ${}^{†}$	9.4 (1.3)	0.277 ${}^{†}$
10-min Apgar score	9.4 (1.5)	9.1 (1.9)	0.432 ${}^{†}$	9.8 (0.4)	0.292 ${}^{†}$
Apnea	11 (11.8)	7 (35.0)	0.012 ${}^{§}$	4 (40.0)	0.041 ${}^{§}$
Hypoglycemia	22 (23.7)	5 (25.0)	0.766 ${}^{*}$	2 (20.0)	1.000 ${}^{§}$
Sepsis	41 (44.1)	11 (55.0)	0.228 ${}^{*}$	7 (70.0)	0.085 ${}^{§}$
Hypothermia	8 (8.6)	2 (10.0)	0.672 ${}^{§}$	0 (0.0)	1.000 ${}^{§}$
Osteopenia of prematurity	4 (4.3)	2 (10.0)	0.261 ${}^{§}$	1 (10.0)	0.414 ${}^{§}$
Hypotension	5 (5.4)	1 (5.0)	1.000 ${}^{§}$	0 (0.0)	1.000 ${}^{§}$
Thrombocytopenia	3 (3.2)	1 (5.0)	0.523 ${}^{§}$	0 (0.0)	1.000 ${}^{§}$
Respiratory distress syndrome	38 (40.9)	10 (50.0)	0.299 ${}^{*}$	5 (50.0)	0.507*
Bronchopulmonary dysplasia	3 (3.2)	3 (15.0)	0.057 ${}^{*}$	1 (10.0)	0.414 ${}^{§}$
Necrotizing enterocolitis	18 (19.4)	2 (10.0)	0.516 ${}^{*}$	1 (10.0)	1.000 ${}^{§}$
Intraventricular hemorrhage	8 (8.6)	5 (25.0)	0.029 ${}^{§}$	2 (20.0)	0.214 ${}^{§}$
Heart disease (including PDA)	23 (24.7)	5 (25.0)	0.844 ${}^{*}$	1 (10.0)	0.441 ${}^{§}$
Hematocrit level	40.5 (6.3)	38.2 (7.3)	0.164 ${}^{†}$	35.1 (4.2)	0.015 ${}^{†}$
Days of total oxygen (days)	6 (2 to 17)	14.5 (6 to 33.5)	0.045 ${}^{‡}$	11.5 (8 to 23)	0.313 ${}^{‡}$
Days of mechanical ventilation (days)	2 (0 to 7)	4 (5 to 8)	0.343 ${}^{‡}$	3 (1 to 6)	0.930 ${}^{‡}$
Days of CPAP (days)	1 (0 to 3)	2 (1 to 5.5)	0.021 ${}^{‡}$	2 (1 to 4)	0.207 ${}^{‡}$
Use of surfactant	15 (16.1)	3 (15.0)	1.000 ${}^{§}$	2 (20.0)	0.789 ${}^{§}$
	
	
white<bcol>6</ecol>ROP, retinopathy of prematurity; PDA, patent ducts arteriosus. ${}^{a}$ Comparison between No ROP and Any ROP; ${}^{b}$ Comparison between ROP requiring intervention and Others (no intervention). ${}^{*}$ Data shown as *n* (%); *P*-value was obtained from Chi-squared test; ${}^{†}$ Data shown as mean (SD); *P*-value was obtained from *t*-test; ${}^{‡}$ Data shown as median (IQR); *P*-value was obtained from Mann–Whitney U test; ${}^{§}$ Data shown as *n* (%); *P*-value was obtained from Fisher's exact test.

**Table 2:** Stepwise multivariable logistic regression – Risk factors for any ROP.


	orange**Odds ratio**	orange**95% CI**	orange ***P*** **-value**
Birth weight	0.997	0.994–0.999	0.005*
Gender: male	4.907	1.079–22.325	0.040*
Bronchopulmonary dysplasia	17.047	1.453–199.946	0.024*
	
	
white<bcol>4</ecol>ROP, retinopathy of prematurity; CI, confidence interval ${}^{*}$ Statistically significant.

**Table 3:** Stepwise multivariable logistic regression – Risk factors for ROP that required intervention.


	orange**Odds ratio**	orange**95% CI**	orange ***P-*** **value**
GA	0.552	0.366–0.830	0.004*
Twin/Triplet	10.530	1.629–68.058	0.013*
	
	
white<bcol>4</ecol>ROP, retinopathy of prematurity; GA, gestational age; CI, confidence interval. ${}^{*}$ Statistically significant.

##  METHODS

This study was a retrospective chart review conducted at Surin Hospital, northeastern Thailand. Surin Hospitals are rural tertiary care hospitals that provide neonatal intensive care units with qualified neonatologists. This study was performed by reviewing the charts of consecutive patients who underwent screening examinations for ROP between October 2019 and September 2020. Ethical review approval was obtained from the Institutional Review Board of Surin Hospital on March 2021 (IRB No. 06/2564). This study was conducted in accordance with the principles of the Declaration of Helsinki. All charts were reviewed by the primary author and no personal data of the patients were disclosed.

The study included all infants who met one of the following criteria: GA ≤ 30 weeks or BW ≤ 1,500 gr; GA > 30 weeks or BW > 1500 gr with an unstable clinical course and believed by their attending pediatrician or neonatologist to be at risk for ROP. These inclusion criteria followed the recommendations of the American Academy of Pediatrics, American Academy of Ophthalmology, and American Association for Pediatric Ophthalmology and Strabismus.^[11]^ Patients lost to follow-up before the screening date were excluded. All examinations were performed by qualified ophthalmologists using binocular indirect ophthalmoscopy. Infants underwent a screening examination at four weeks postnatal age or a corrected GA of 31 weeks, whichever occurred later. Retinal findings and ROP stages were recorded according to the revised International Classification of Retinopathy of Prematurity.^[12]^ The intervals of subsequent examinations were determined by the examining ophthalmologist in accordance with the severity of the findings. Interventions were performed when ROP reached type-1 pre-threshold disease or a diagnosis of aggressive posterior retinopathy of prematurity (APROP) was noted.

The primary outcome was to estimate the incidence of any stage of ROP (stages I–V) and ROP requiring intervention. The identification of risk factors for the development of any ROP and ROP requiring intervention was the secondary outcome. The variables collected were as follows:

(1) Demographic information: sex, BW, and multiple gestations (twins or triplets).

(2) Antenatal factors: GA, maternal age, number of gestations, inadequate number of antenatal care (ANC) visits (less than four visits), maternal diabetes mellitus, premature rupture of membranes, and severe preeclampsia.

(3) Neonatal factors: Apgar scores at 1, 5, and 10 min, apnea of prematurity, hypoglycemia, neonatal sepsis (culture-positive or antibiotic administration for more than seven days), hypothermia, osteopenia of prematurity, hypotension, thrombocytopenia, RDS, BPD, necrotizing enterocolitis, IVH, heart disease including patent ductus arteriosus, hematocrit level (on the date of discharge), use of surfactant, total days of oxygen therapy, days of mechanical ventilation, and days of continuous positive airway pressure (CPAP).

### Statistical Analysis

All analyses were performed using Stata 13.0 (StataCorp, College Station, TX, USA). In the antenatal factor analysis, only one participant with multiple pregnancies was randomly selected to eliminate duplication of the data. Univariable comparisons of risk factors between the groups were analyzed. For variables with univariable *P*-value < 0.2, forward stepwise multivariable logistic regression was then used to establish the association between risk factors and the development of any ROP and ROP requiring intervention. The significance level for adding a variable was set as 0.05. Demographic, antenatal, and neonatal factors were explored using a stepwise model. Regression analysis was performed to define associations. Odds ratios (ORs) and 95% confidence intervals (CIs) were also calculated. Continuous variables are shown as means with standard deviations (SD) or medians with interquartile ranges. Categorical variables were presented as counts and percentages. Continuous data were analyzed using the *t*-test or Mann–Whitney U test depending on the distribution of data. Categorical data were analyzed using the Chi-squared test or Fisher's exact test, as appropriate. For all tests, a *P*-value < 0.05 was considered statistically significant.

##  RESULTS

During the study period, 119 infants met the criteria for the ROP examination. Of the 119 infants, 40 (33.6%) had a GA of 30 weeks or below, and 53 (44.5%) had a BW of 1500 gr or below. Thirty-three infants (27.7%) had a GA ≤ 30 weeks and BW ≤ 1500 gr. A total of 59 infants (49.6%) had a GA > 30 weeks and BW > 1500 gr. These infants underwent ROP screening because of an unstable clinical course, including RDS in 18 infants, pneumonia in 16 infants, and sepsis in 25 infants. Of these, six patients were lost to follow-up before the date of screening; thus, they were excluded. Subsequently, 113 participants underwent ROP screening and were included in the present study [Figure 1]. Of the no ROP-diagnosed infants, subsequent examinations were performed every 4 weeks until a postmenstrual age of at least 45 weeks, averaging 3.5 examinations. Of the ROP-diagnosed infants, subsequent examinations were performed every 2 weeks until a postmenstrual age of at least 54 weeks and were examined until total regression of ROP was observed, which averaged approximately 12.3 examinations.

The incidence rate of ROP was 17.7% (20/113). Of the ROP-diagnosed patients, 40% (8/20), 10% (2/20), 15% (3/20), and 5% (1/20) developed stages 1, 2, 3, and 4 ROP, respectively, and 30% (6/20) had APROP. None of them developed stage 5 ROP. Meanwhile, the incidence of ROP requiring intervention was 8.8% (10/113) among all infants undergoing the examination and 50% (10/20) of known patients with ROP. All patients with ROP who required intervention achieved satisfactory anatomical outcomes after treatment.

Table 1 shows the results of the univariate analysis. Infants with any stage of ROP had significantly lower GA, lighter BW, higher incidence of IVH, higher incidence of apnea, longer duration of total supplemental oxygen, and longer duration of CPAP than infants without ROP. The mean GA and BW of infants with ROP were 30 ± 3.2 weeks and 1294 ± 246.8 gr, respectively. While the mean GA and the mean BW of infants without ROP were 32.2 ± 2.9 weeks and 1656.1 ± 500.9 gr, respectively. Using stepwise multivariable logistic regression [Table 2], the following were significant risk factors for ROP development: lighter BW (OR 0.997, 95% CI 0.994–0.999, *P* = 0.005), male gender (OR 4.907, 95% CI 1.079–22.325, *P* = 0.040), and BPD (OR 17.047, 95% CI 1.453–199.946, *P* = 0.024). Lower GA (OR 0.552, 95% CI 0.366–0.830, *P* = 0.004) and being a twin/triplet (OR 10.530, 95% CI 1.629–68.058, *P* = 0.013) were significant risk factors for the development of ROP requiring intervention as shown in the multivariable logistic regression analysis [Table 3]. No antenatal factor was identified as a predictor or risk factor of ROP development.

##  DISCUSSION

The incidence of ROP at any stage varies among countries, with reported figures ranging from 18.5% to 47%.^[3,8,9]^ This was comparable to the result of Hong Kong's study (any ROP incidence of 18.5%).^[9]^ In Thailand, there are few published studies on the incidence of ROP. A large study from an urban tertiary hospital in Bangkok revealed that the incidence of ROP during the last decade (2006–2009) was 40.7%; among these, 72% required intervention.^[10]^ More recently, in 2020, a study conducted by another urban tertiary hospital showed that the incidence of ROP and ROP requiring intervention was 10% and 3%, respectively.^[13]^ The current incidence of ROP reported by rural tertiary hospitals ranges from 20.1 to 31.7%, and that of ROP requiring intervention is 10.8%.^[14,15]^ It signifies that the incidence of ROP varies considerably among local literature and this may be a reflection of the modification of ROP screening criteria and the improvement of antenatal and neonatal cares over the past decade. Additionally, evidence shows that the incidence of ROP is higher in rural than in urban tertiary hospitals. The differences in specific risk factors, particularly the characteristics of neonatal care, could play a role in the disparity in the ROP incidence between these two areas.

To the best of our knowledge, lower GA and lighter BW are widely regarded as the major risk factors for ROP development.^[5]^ Our univariable analysis showed that infants with any stage of ROP had significantly lower GA and lighter BW than those without ROP; however, only lighter BW was statistically significant in the multivariable logistic regression model. Similarly, a recent Korean nationwide population-based study demonstrated that the incidence of ROP (317.14 per 1000 newborns) and the rate of visual impairment (4.5 per 100 person-years) were the highest among very low BW infants.^[16]^ Moreover, lower GA was the significant risk factor for both ROP and ROP requiring intervention, in stepwise analysis. This finding is consistent with the results of most studies.^[4,17,18,19]^

Male gender was the other risk factor for any ROP development in this study. Yang et al also found a similar relationship in their analysis.^[20]^ One possible explanation is male vulnerability. It has been described that morbidity and mortality are frequently reported to be higher in male gender than female gender in early life.^[21]^

BPD was one of the important risk factors for any stage of ROP in this study. Our result was in line with two previous local studies.^[13,15]^ Gebeşçe et al, Holmström et al, and Wu et al also found a similar relationship in multivariable analysis.^[22,23,24]^ Furthermore, Singh and colleagues revealed the association between moderate-to-severe BPD and severe ROP.^[6]^ Interestingly, it has been proposed that both BPD and ROP may share common molecular mechanisms predisposing to dysregulation of angiogenesis.^[25]^

Being a twin or triplet was significantly correlated with the development of ROP requiring intervention in our study; however, this condition was not associated with any ROP development. Likewise, Port and colleagues identified multiple gestations as a risk factor for treatment requiring ROP.^[26]^ There is evidence that twins could exist in placental-sharing nutrition and blood supply situations, which may reduce BW discordance.^[27]^ It is well-known that the proportion of infants with extremely low BW is greater in twins than in singletons.^[28]^ The extreme low BW has been frequently identified as a major risk factor for ROP requiring intervention.^[8]^

The present study has some limitations that should be considered. This was a single-center retrospective study. Further multicenter studies with larger sample sizes are still needed to provide generalizable epidemiological data on ROP in Thailand.

In summary, the incidence of ROP at any stage and ROP requiring intervention was 17.7% and 8.8%, respectively, at a rural tertiary hospital in Thailand. Lighter BW, male gender, and BPD were significant risk factors for the development of any ROP. Lower GA and being a twin or triplet were other relevant conditions affecting ROP requiring intervention. As the survival rates of preterm infants are increasing annually, investigation of the incidence of ROP and its risk factors is critical for identifying potential hotspots for ROP and establishing a screening protocol to prevent ophthalmic morbidities. Guidelines must be updated in a local context, and further epidemiological studies are imperative.

##  Financial Support and Sponsorship

The authors did not receive support from any organization for the submitted work.

##  Conflicts of Interest

The authors have no relevant financial or non-financial interests to disclose.
